# Supplementary material for: Drinking to Cope Mediates the Association between Dyadic Conflict and Drinking Behavior: A Study of Romantic Couples during the COVID-19 Pandemic
Source: Int J Environ Res Public Health. 2023 Jul 10;20(14):6332. doi: 10.3390/ijerph20146332 (PMC10379069; doi:10.3390/ijerph20146332)
Supplement: Supplementary file 1 [file ijerph-20-06332-s001.zip › ijerph-2455086-supplementary.pdf]

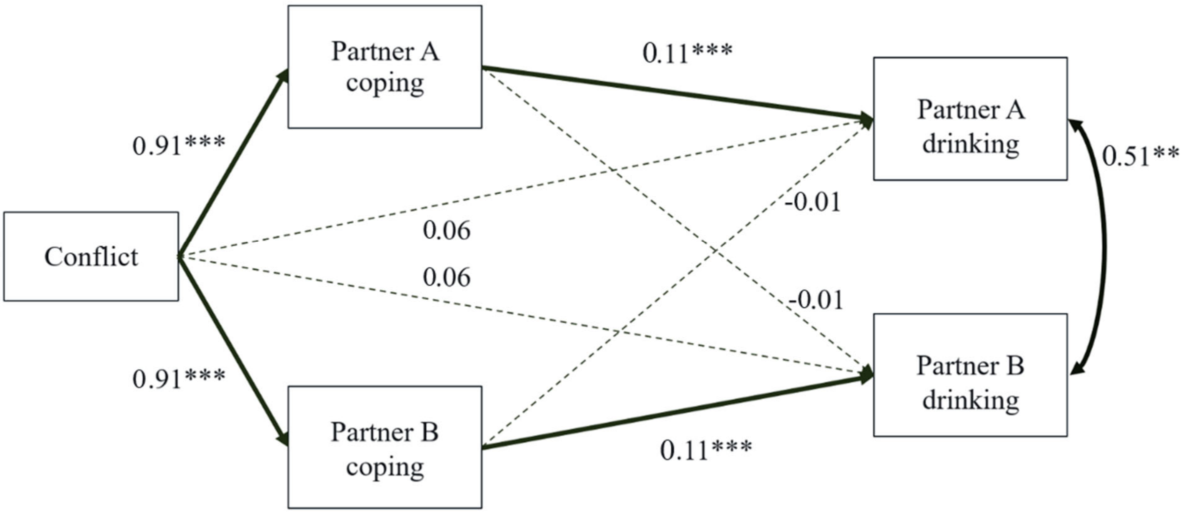

Figure S1. Generalized SEM Model with Indistinguishable Dyads controlling for income.

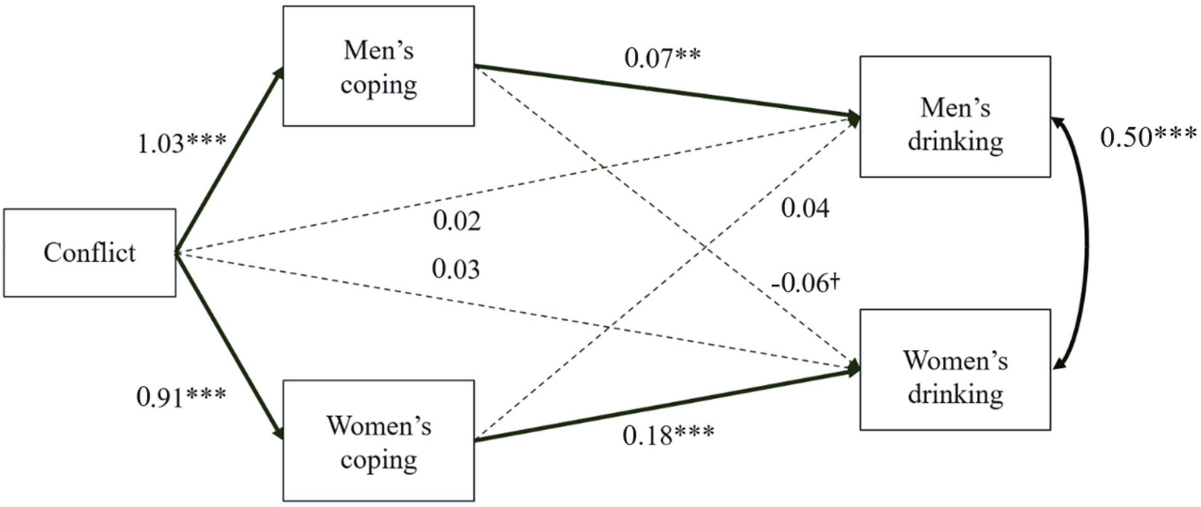

Figure S2. Generalized SEM Model with Distinguishable Dyads controlling for income

Table S1. Direct and indirect effects of conflict on drinking in indistinguishable dyads controlling for income.

Effect	Path	*b*	*SE*	*p*	IRR	LCI	UCI
Direct	Conflict → A Drinking	0.06	0.03	0.063	1.06	1.00	1.13
Indirect	Conflict → A Coping → A Drinking	0.10	0.02	<0.001	1.11	1.07	1.15
Indirect	Conflict → P Coping → A Drinking	-0.01	0.02	0.437	0.99	0.96	1.02
Total	Conflict → A Drinking	0.15	0.02	<0.001	1.16	1.11	1.22

Note. IRR = Incident Rate Ratio. LCI and HCI are lower and upper 95% confidence intervals from bias-corrected bootstrapping with 1000 resamples. A = actor; P = partner. *n = 327.*

Table S2. Direct and indirect effects of conflict on men and women partners' coping and drinking controlling for income.

Effect	Path	*b*	*SE*	*p*	IRR	LCI	UCI
Direct	Conflict → M Drinking	0.02	0.03	0.645	1.02	0.95	1.08
Indirect	Conflict → M Coping → M Drinking	0.07	0.03	0.005	1.07	1.02	1.13
Indirect	Conflict → W Coping → M Drinking	0.04	0.02	0.111	1.04	0.99	1.09
Total	Conflict → M Drinking	0.12	0.03	<0.001	1.13	1.07	1.20
Direct	Conflict → W Drinking	0.03	0.04	0.542	1.03	0.94	1.12
Indirect	Conflict → W Coping → W Drinking	0.16	0.03	<0.001	1.17	1.11	1.26
Indirect	Conflict → M Coping → W Drinking	-0.06	0.03	0.048	0.94	0.89	1.00
Total	Conflict → W Drinking	0.13	0.03	<0.001	1.14	1.07	1.21

Note. IRR = Incident Rate Ratio. LCI and HCI are lower and upper 95% confidence intervals from bias-corrected bootstrapping with 1000 resamples. *n = 289.* M = men; W = women.
